# Impact of COVID-19 in AChR Myasthenia Gravis and the Safety of Vaccines: Data from an Italian Cohort

**DOI:** 10.3390/neurolint14020033

**Published:** 2022-04-27

**Authors:** Antonino Lupica, Vincenzo Di Stefano, Salvatore Iacono, Antonia Pignolo, Martina Quartana, Andrea Gagliardo, Brigida Fierro, Filippo Brighina

**Affiliations:** Section of Neurology, Department of Biomedicine, Neuroscience and Advanced Diagnostics (BIND), University of Palermo, 9012 Palermo, Italy; antlupica@gmail.com (A.L.); salvo.iak@gmail.com (S.I.); niettapignolo@gmail.com (A.P.); quartanamartina@gmail.com (M.Q.); andrigl@gmail.com (A.G.); brigida.fierro@unipa.it (B.F.); filippobrighina@gmail.com (F.B.)

**Keywords:** COVID-19, SARS-CoV2, vaccine, Myasthenia gravis, neuromuscular, myasthenic crisis

## Abstract

Background and aims. Patients with Myasthenia gravis (MG) are considered vulnerable as they may present with respiratory muscle weakness and because they are on immunosuppressive treatment; thereby, COVID-19 may have a detrimental effect on these patients. Vaccines against COVID-19 are currently available and it has been shown as they can prevent severe COVID-19 in vulnerable patients. Notwithstanding their efficacy, vaccine hesitancy has not been completely dispelled in the general population. Unfortunately, there is limited data about the safety of these vaccines in MG patients. The aims of this study are to evaluate the impact of COVID-19 in a MG cohort, the adherence to COVID-19 vaccination in Italy and vaccine safety in MG patients. Methods. A retrospective cohort study of MG patients attending the Neuromuscular Clinic of the University Hospital “Paolo Giaccone” of Palermo, Italy, was performed. Patients underwent telephone interviews with a dedicated questionnaire on SARS-CoV-2 vaccination and infection. Vaccine safety was assessed though the evaluation of vaccine-related adverse events (AEs) and comparisons of MG-ADL scores before and after vaccination. Patient worsening was defined as two or more point increases in MG-ADL scores. Results. From a total of 90 participants, 75 answered the questionnaire and 70.5% of them (n = 53) received the vaccine; ten patients did not receive vaccination and 3 patients were partially vaccinated. Among the vaccinated patients, about 45% (n = 24) experienced at least one AE, with a complete resolution within one week. No serious AEs and life-threatening conditions were observed. Globally, MG-ADL scores did not worsen after vaccination. Nine unvaccinated patients experienced SARS-CoV2 infection and four of them (44%) died—one patient required respiratory support, whereas three patients were asymptomatic. Conclusions. COVID-19 significantly impacted MG patients with an increase in mortality due to respiratory sequelae. Vaccines against SARS-CoV-2 showed good short-term safety in MG patients, who may take advantage of vaccination to avoiding life-threatening complications such as COVID-19 pneumonia.

## 1. Introduction

Myasthenia gravis (MG) is a chronic autoimmune disorder of the neuromuscular junction characterized by autoantibodies against acetylcholine receptors (AChR-Ab), muscle-specific kinase (MuSK-Ab), lipoprotein-related protein 4, or agrin in the postsynaptic membrane at the neuromuscular junction [1]. Sometimes this condition requires hospitalization for potentially lethal exacerbations that may be triggered by infections, stress, and vaccines [2,3]. Hence, the recent SARS-CoV-2 outbreak has led to several critical issues in the management of MG patients [4,5,6,7]. The first concern arises because many drugs used in the first phase of the Coronavirus Disease 2019 (COVID-19) pandemic (e.g., azithromycin, chloroquine, and hydroxychloroquine) are contraindicated in MG [8,9], with detrimental effects on these patients—there can be clinical worsening until myasthenic crisis in such cases [10]. Additionally, the broader use of anesthetics, intubation, and mechanical ventilation in COVID-19 patients hospitalized in Intensive Care Units might have led to additional risks for patients with generalized MG [8]. On the contrary, immunosuppressant drugs usually protect from MG exacerbation and worse COVID-19 outcomes in MG patients [11]. Additionally, the double face of immunosuppression has raised different opinions on the management of MG patients with COVID-19. Consequently, many reports have been published describing MG onset or MG exacerbations after SARS-CoV 2 infection [7,11,12,13,14,15,16,17,18,19]. Recent studies and editorials have appeared giving suggestions for the management of MG during the SARS-CoV-2 pandemic [5,8,12], but there are still few studies from real life exploring the impact of COVID-19 in MG [4].

Moreover, after the first wave of the pandemic, vaccines against COVID-19 were developed and preliminary data have showed a good efficacy and safety profile [13]. However, there are a few studies exploring the impact of vaccines against SARS-CoV-2 in MG. This topic has already been discussed in some editorials supporting COVID-19 vaccination in NMD patients, but there is again a lack of data from MG patients [14].

Indeed, some viral-derived products—including SARS-CoV2 antigens—may have a role as triggers through natural infection and, less commonly, after vaccination [15,16]. Several mechanisms, including molecular mimicry and the use of adjuvants, may be responsible for these phenomena [16,17,18,19,20,21]. The immunogenicity of the available SARS-CoV-2 vaccines may depend on the intrinsic adjuvanticity of mRNA or DNA vaccines which, respectively, are able to stimulate innate immunity through Toll-Like Receptors 3, 7, 8, and 9, and components of the inflammasome [16,18,22,23,24,25].

Furthermore, randomized and controlled trials (RCTs) exploring the safety and efficacy of vaccines are not generally performed on subjects with neuromuscular disorders (NMD), including MG [16,26,27,28]. Moreover, the vaccine’s safety is generally considered a secondary endpoint, with pivotal RCTs being underpowered to enable statistical analyses of endpoints of specific side effects, including NMD induction or worsening—this is particular true during pandemic periods [16,29]. Hence, there is widespread concern for the safety of SARS-CoV-2 vaccines in patients affected by autoimmune diseases such as MG.

In this study, we examined the impact of COVID-19 in an Italian cohort of MG patients and we evaluated the rate of adherence to the Italian vaccination program against SARS-CoV-2 while investigating the tolerability of vaccines against COVID-19.

## 2. Materials and Methods

### 2.1. Study Population

We performed a retrospective cohort study in a cohort of patients with MG attending the Neuromuscular Clinic of the University Hospital “Paolo Giaccone” of Palermo in the period between May and October 2021. Inclusion criteria were age > 18 years, signed informed consent, and diagnosis of MG according to existing recommendations (i.e., at least two diagnostic tests suggestive of MG) [27]. Patients that had not given informed consent or with incomplete diagnostic workup for MG were excluded.

### 2.2. Questionnaire

The participants underwent a telephone interview with a dedicated questionnaire containing questions about vaccination against SARS-CoV-2, type of vaccine administrated, date of first and second doses, number of vaccine doses, and presence (i.e., after first, second or after both doses) and types (i.e., local pain, asthenia, cephalalgia, fever, myalgia,) of vaccine-related adverse events (AEs). The term AE used in this study indicates transient local or systemic reactogenicity following vaccination, with a resolution within one week. Additionally, questions on SARS-CoV-2 infection (i.e., severity, outcomes, therapy) were included in the questionnaire.

### 2.3. MG Outcomes

The MG-ADL (Myasthenia Gravis Activity Day Living) [30,31] score was employed to estimate the quality of life in MG patients; MG-ADL scores were collected before and after vaccination for each patient. The score before vaccination was available for each patient from previous neurological examination, whereas MG-ADL scores after vaccination were collected through telephone interviews. Patients after vaccination were defined as “stable” if the variation in MG-ADL score was 0–1 points, while a variation ±2 was considered as “worsening” (+2) or “improving” (−2) [32]. The variation in MG-ADL score collected within 3 weeks after vaccination and the relationship between vaccine AEs and MG-ADL outcomes (worsened, stable, improved), type of vaccine administered, gender, age, type of MG, and type of serum antibody were evaluated in the participants. Finally, we compared demographic characteristics between MG patients who died because of COVID-19 and patients who survived.

### 2.4. Statistical Analysis

The qualitative (i.e., demographic data, MG history data, type of vaccine, AEs frequency) and quantitative variables (i.e., age, MG-ADL score) were reported as absolute, with relative frequency and median with interquartile range (IQR), respectively. Differences in MG-ADL scores between groups (group 1: MG patients before vaccination; group 2: MG patients after vaccination) were estimated using the Wilcoxon signed rank test. Conversely, the distribution of the qualitative variables among the MG patients was evaluated using the Chi-squared test. Continuous variables (i.e., age, antibody titers) were compared using the Kruskal–Wallis test. The statistical analyses were performed using SPSS software (version 26.0 IBM Statistics, IBM Corp. Lane Cove, NSW, Australia); the level of significance was set at *p* value < 0.05.

## 3. Results

**Figure 1:**
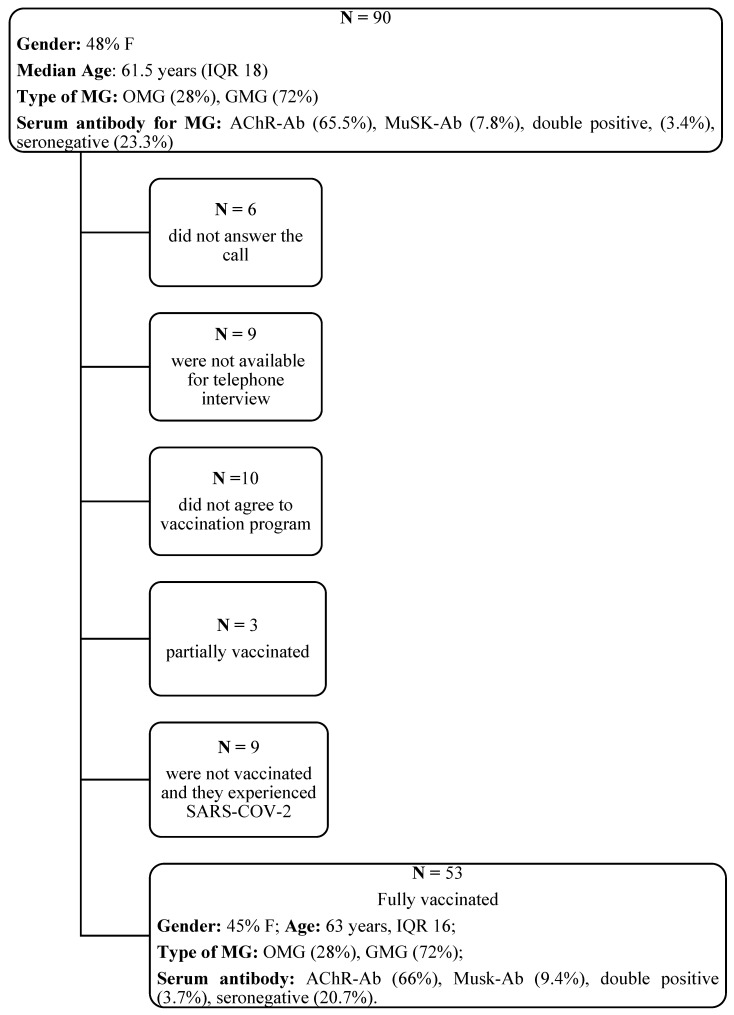
Flow-chart of recruitment procedure and demographic features of study participants. OMG, Ocular Myasthenia Gravis; GMG, Generalized Myasthenia Gravis; F, female; AchR-ab, acetylcholine receptors; MuSK-Ab, muscle-specific kinase.

A total of 90 patients with MG (48% females, median 61.5 years, IQR 18) with regular six-monthly follow-ups were enrolled during the study period. Figure 1 summarizes the recruitment procedure.

**Figure 2:**
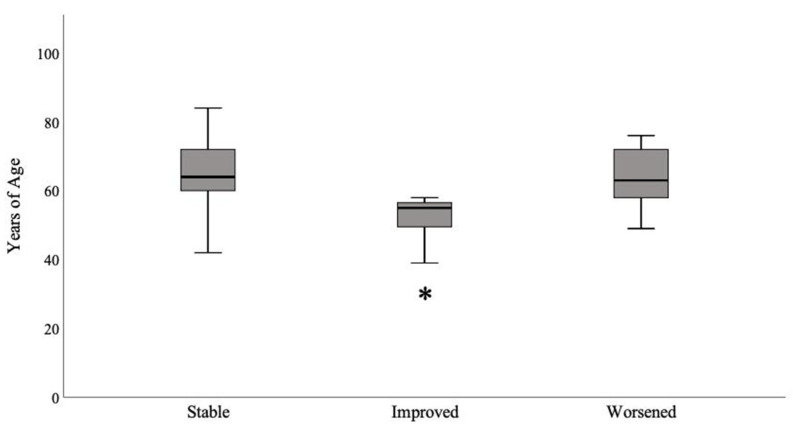
Box-plots showing the median age according to MG-ADL outcomes. Patients with MG-ADL improvement were younger compared to others (* *p* = 0.03). Median and interquartile ranges are reported.

Fifteen patients (16.5%) were excluded from the analyses because they did not answer the questionnaire; therefore, 75 patients were in the final study sample. Thirteen percent of patients (n = 10) did not receive the vaccination, whereas 4% (n = 3) received only the first dose—they were excluded from the analysis. Nine patients (12%) experienced SARS-CoV-2 infection and were not vaccinated at the time of the interview. A total of 70.5% of the patients (n = 53; 45% females, median age 63 years, IQR 16) completed the vaccination program. Ninety percent of patients (n = 48; 48% females; median age 63 years, IQR 14) received BNT162b2, whereas 7.5% (n = 4, 25% females; median age 74 years, IQR 22) received mRNA-1273, and only one male patient received ChAdOx1 nCoV-19 (AZD1222, AstraZeneca). The median times between the first and second doses and the interview were 31.5 days (IQR 18) and 7 days (IQR 20), respectively. The MG-ADL score after vaccination remained stable in 58.5% of patients (n = 31, 32% females), improved in 15% (n = 8, 50% females), and worsened in 28.3% of patients (n = 14, 71% females). Overall, the MG-ADL score after vaccination did not differ compared to the one obtained before vaccination (median 3, IQR 4 vs. median 3, IQR 5; *p* = 0.2). MG-ADL outcomes were not related to type of vaccine administrated, type of MG, presence and types of AEs after first and second dose, or with serum MG antibodies (overall *p* > 0.05). The median titers of serum AchR-ab were similar between vaccinated and unvaccinated patients (7.3 vs. 5.9 nmol /L; *p* = 0.56), as well as serum MuSK-ab titers (25.1 vs. 31 U/mL; *p* = 1). Median titers of AchR-ab were not different among stable (18 nmol/L, IQR 37), improved (8 nmol/L, IQR 18), and worsened (8 nmol/L, IQR 99) patients (*p* = 0.4). MG-ADL worsening was more common in females compared to males (71% vs. 32%; *p* = 0.048). Of note, patients with MG-ADL improvement were younger compared to stable (*p* = 0.008) and worsened patients (*p* = 0.03; Figure 2).

Table 1 summarize the demographic and clinical features of patients with MG-ADL score worsening after vaccination.

**Figure 3:**
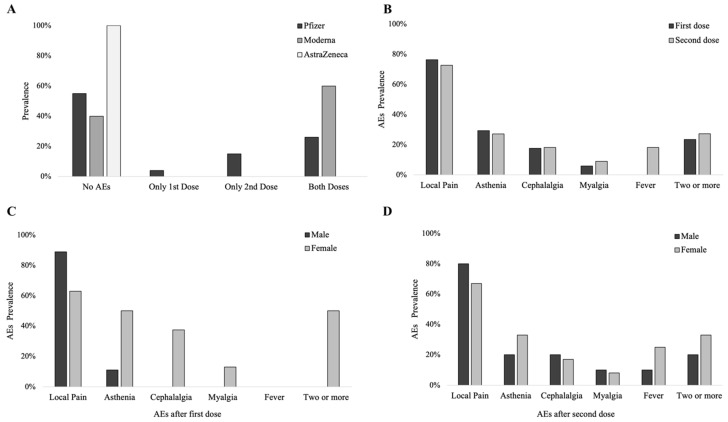
(**A**) Percentage of patients presenting with AEs after vaccination (vertical axis), reported according to vaccine type. (**B**) AE prevalence after first and second dose. (**C**,**D**) AE prevalence after first (**C**) and second dose (**D**), according to gender.

A total of 54.7% of the vaccinated patients (n = 29) did not experience AEs after vaccine administration, whereas 3.8% (n = 2), the 13.2% (n = 7) and 28.3% (n = 15) of patients reported AEs after the first, second, or both doses, respectively. Patients who received Pfizer experienced AEs after only the first or second doses and more commonly after both doses, whereas those who received AstraZeneca did not experience any AEs; patients who received Moderna principally experienced AEs after both doses (Figure 3A).

Among the patients reporting AEs, 54.2% were female, whereas 45.8% were male, without significant differences between groups. After the first dose, 17 patients (70.8%) experienced AEs, which consisted of local pain (76.5%, n = 13), asthenia (29.4%, n = 5), cephalalgia (17.6%, n = 3), myalgia (5.9%, n = 1), and with four patients (23.5%) experiencing two or more AEs (Figure 3B). No patients experienced fever after the first dose. A total of 22 patients (91.6%) experienced AEs after the second dose, with local pain being the most common AE (72.7%, n = 16), followed by asthenia (27.2%, n = 6) and fever (18.2%, n = 4). The occurrence of AEs was not significantly associated with gender, age, type of MG, MG-ADL outcomes, type of serum antibody, disease onset after or before 60 years of age, and type of vaccine received. AchR-ab median levels did not differ in patients with (6.3 nmol/L, IQR 19) and without (14 nmol/L, IQR 16) AEs (*p* = 0.4), as well as MuSK-ab levels (*p* = 1). Of interest, females experienced more AEs compared to males after the first dose (*p* = 0.04; Figure 3C), with local pain being the only exception—being more common in males (not significant, *p* = 0.09). After the second dose, the types of AEs that occurred were similar between groups (Figure 3D).

### Impact of COVID-19 in MG Patients

In our cohort, nine patients (12%; 4 females; median age 66 years, IQR 41) tested positive for SARS-CoV2 infection, as confirmed by polymerase chain reaction. All these patients were not vaccinated. Five patients (56%) were symptomatic for COVID-19; among them, four patients (80%) died with COVID-19, whereas one patient was hospitalized in an intensive care unit (ICU), requiring mechanical respiratory support. Three patients (33%) were asymptomatic and underwent diagnostic molecular analysis for SARS-CoV-2 because they were exposed to the virus, whereas one patient (11%) experienced flu-like symptoms lasting for five days (Table 2).

The deceased patients experienced MG onset after 60 years of age more often compared to those who survived (*p* = 0.008), and they were significantly older (median age 42 years, IQR 32 vs. median age 76.5, IQR 15; *p* = 0.03). There were not significant differences between deceased and survived patients depending on gender, MG serum antibody, or MG-ADL score. However, deceased patients took more medications and presented with a higher number of comorbidities compared to patients who survived COVID-19, even if these results did not reach the statistical significance. Of note, the presence of diabetes was significantly more common in deceased patients (*p* = 0.04).

## 4. Discussion

The burden of COVID-19 in MG patients over the last two years has been the object of a few case series and retrospective studies [4,33,34], demonstrating a clinical deterioration of MG patients and going as far as admission to the ICU in more severe cases [11,35,36,37,38]. These studies reported up to 30% lethality [4,33,36,37,39] and a high rate of complications [33,34,36,37,38]. The prevalence of COVID-19 is broadly variable between population studies, from between 0.96 to 30%, with data heterogeneity reflecting several factors—including diffusion of SARS-CoV2, the period of study, and vaccination policies [40]. In this paper, we aimed to provide an overview of the impact of COVID-19 and vaccination programs in a tertiary-referral MG Centre. At the very beginning, in the first wave of the pandemic, worldwide social restrictions were the only solution for slowing the diffusion and the rapid spread of COVID-19 [41]. In a first study published during the first wave, we explored the impact of lockdown on patients affected by NMD [7]; as expected, the restrictions significantly reduced the patients’ overall autonomy and some patients refused clinical follow-up. NMD patients were afraid of COVID-19—this was a limiting factor for clinical assessments and follow-up. However, taken together, fear and social restrictions seem to have also contributed to reducing the risk of infection in MG patients [5,8]; indeed, many centres and neurologists adopted telemedicine and telephone consultations in their clinics [42,43]. As a consequence, only about 10% of patients in our population were affected by COVID-19. In this particular setting, in our centre, a telephone interview allowed clinicians to reduce the infective risk, while administering simple questionnaires and scales such as the MGADL.

Our data on infected patients showed that COVID-19 was lethal in 44% of MG patients infected and that it required hospitalization in 55%. It is already known that MG patients are prone to develop lethal COVID-19 [4]—especially patients with oncologic and autoimmune comorbidities [33]. In fact, the respiratory involvement in COVID-19 may aggravate the pre-existing respiratory disorder in MG patients due to the peculiar weakness of the diaphragmatic muscles that exposes such patients to the development of dyspnea and early fatigue of the respiratory muscles, and, in more severe cases, may cause a muscle pump deficit incompatible with life. Hence, generalized MG patients require special respiratory support with COVID-19 infection. Moreover, in our cohort, patients who died due to COVID-19 were older, with a higher age at disease onset, and they had more comorbidities (diabetes). According to the Italian vaccination program, these patients received a priority to get timely vaccinations.

Vaccines against COVID-19 have already shown a good safety and efficacy in the general population. The most common AEs after BNT162b2 and mRNA-1273 vaccination are local pain (66–83%), followed by asthenia (51–59%), cephalalgia (25–52%), myalgia (19–37%), and gastrointestinal symptoms (8–12%), which are more common after the second dose and in younger subjects [44,45]. After ChAdOx1 nCoV-19, a higher prevalence of asthenia has been noted (70%), together with myalgia (60%) and fever (18%)—with a marked reduction in AE_s_ after premedication with paracetamol [46]. Conversely, in our population, only minor AEs were observed after vaccination, in accordance with the literature [44,45,47]. Taken together, our data showed the reliability and high safety profile of COVID-19 mRNA BNT162b2 (Pfizer-BioNTech). Unfortunately, only four patients received Spikevax mRNA-1273 (Moderna) and only one patient ChAdOx1 nCoV-19 (AZD1222, AstraZeneca, Cambridge, UK); hence, it was not possible to compare data among different types of vaccines. Moreover, as exacerbation or clinical worsening might be expected in MG patients, we decided to compare MG-ADL scores before and after the administration of vaccines to underline possible variations following vaccination in MG patients. The principal finding was a stable MG-ADL score after vaccination, with no significant variations in our cohort. Furthermore, we found a worsening of the MG-ADL score in only a few patients (Table 2). The MG-ADL score increased in patients who had required rescue therapies because of incomplete control of their symptoms (patients 1, 2 and 7); in these cases, the increase in the MG-ADL score may have been just a coincidence, independent of vaccine administration. Similarly, patient 3 received low doses of CTS because he refused other treatments. Conversely, patients 8 and 13 were on steroid withdrawal in the previous two months—thus justifying the clinical worsening. Patient 9 was recently diagnosed with chronic obstructive pulmonary disease (COPD) at the time of vaccination; COPD alone might explain the exacerbation of respiratory symptoms even in absence of a real connection with MG; a similar consideration could be made for patient 4, for whom a radiculopathy may have caused the weakness in their lower limbs.

Further data needs clarification: for example, ocular symptoms in MG are very frequently prone to fluctuations in the myasthenic patient population—even in the presence of good pharmacological compensation. Furthermore, the effect of temperatures in MG should be taken into account, as it is a well-known fact that heat can exacerbate MG. Indeed, the study was conducted in Spring and Summer, when the increasing hot weather, typical of the South of Italy in this period of the year (until 45 °C in July), could have triggered—at least partially—the clinical worsening in MG patients. Unfortunately, it is not possible to make a comparison with similar periods of previous years due to the lack of data. Finally, the “generalized asthenia” that some patients reported may be a misleading symptom in MG, not necessarily being related to MG. Indeed, asthenia is an unspecific symptom in MG present in many other physiological as well as pathological conditions, which can lead to a decrease in physical and mental abilities, reduced resistance to effort, and easy fatigue after the normal activities of everyday life. This symptom, cardinal in MG, was reported in large percentages of the healthy population at the time of vaccination; therefore, it is difficult to consider asthenia as an indicator of clinical deterioration in MG patients. However, it should be noted that this study refers to a period of follow-up within three weeks following vaccination, which, although short, allowed us to have an overall view of the short-term side effects. The long-term effects of vaccines should be explored in further studies with a longer observation period.

## 5. Conclusions

COVID-19 vaccines represent a safe weapon to counteract SARS-CoV-2 infection, even in the myasthenic population. Prevention measures are essential in a pandemic—especially for infections and possible related complications. However, these complications may cause a long hospitalization in myasthenic patients, often being detrimental, with significant repercussions on recovery and quality of life. An accurate evaluation of the advantages and risks of COVID-19 vaccines is needed, but our data support the use of vaccines in MG patients, even in the presence of active immunosuppressive and immunomodulating regimens. Additionally, the low prevalence of AEs in our population suggests that myasthenic patients could take advantage of anti-COVID-19 vaccination, avoiding life-threatening complications such as myasthenic crisis and COVID-19 pneumonia. A more prolonged follow-up, together with serological and immunological data, are needed to investigate the long-term safety and efficacy of COVID-19 vaccines in the myasthenic population.

## Figures and Tables

**Table 1:** Clinical features of patients worsened at MGADL score after vaccination. AB, serum antibody specific for MG; AchR-ab, acetylcholine receptors; MuSK-Ab, muscle-specific kinase; MGADL, Myasthenia Gravis Activities Daily Living; CTS, corticosteroids; IS, immunosuppressant; IVIg, intravenous immunoglobulin; COPD, chronic obstructive pulmonary disease; OSAS, Obstructive sleep apnea syndrome. * Therapy variation in the last year.

Pt	Sex	AB	MGADL Pre–Post	ΔMGADL	Δ Item MGADL	Therapy Variation *	Comorbidity
1	M	MuSK	5–7	2	+1 arise from a chair +1 double vision	IVIg	OSAS
2	F	AChR	2–4	2	+1 double vision +1 swallowing	IVIg	Cataract
3	F	AChR	1–6	5	+2 eyelid droop +1 breathing +2 arise from a chair	No CTS, refuse IS	Vertebral osteoporosis
4	M	AChR	0–3	3	+2 brush teeth or comb hair +1 eyelid droop		Obesity, Radiculopathy
5	F	/	3–6	3	+2 brush teeth or comb hair +1 talking		
6	M	/	2–4	2	+1 eyelid droop +1 breathing		
7	F	AChR	9–11	2	+1 chewing +1 breathing	IVIg	
8	F	AChR	3–6	3	+1 swallowing +2 brush teeth or comb hair	CTS reduction	
9	F	AChR	8–13	5	+2 swallowing +2 chewing +1 breathing		COPD
10	M	AChR	1–3	2	+2 arise from a chair		
11	F	AChR	5–8	3	+2 arise from a chair +1 brush teeth or comb hair		
12	F	AChR	0–7	7	+2 swallowing +2 brush teeth or comb hair +3 eyelid droop		
13	F	/	5–7	2	+1 arise from a chair +1 brush teeth or comb hair	CTS reduction	
14	F	/	1–3	2	+1 breathing +1 eyelid drop		

**Table 2:** Demographic characteristics and medical history of MG patients with SARS-CoV2 infections. MGADL, Myasthenia Gravis Activities Daily Living; AchR-ab, acetylcholine receptors; MuSK-Ab, muscle-specific kinase; VHL, Von Hippel Lindau Syndrome; NIV, non-invasive ventilation.

Pt	Sex	Antibody Specificity	Comorbidity	MGADL before COVID-19	MGADL after COVID-19	Outcome
1	M	AChR	Diabetes	5		Died
2	M	AChR	Diabetes	1		Died
3	F	AChR	Diabetes, chronic renal failure, hypertension	6		Died
4	M	AChR		4	11	Recovered after NIV
5	F	AChR	Diabetes	1		Died
6	M	MuSK, AChR		0	0	Asymptomatic
7	F	AChR		8	8	Asymptomatic
8	M	AChR	//	4	4	Asymptomatic
9	F	AChR	VHL, vertebral hemangioma, migraine, hyperinsulinism, factor XII deficiency	5	5	Recovered after flu-like symptoms (fever, cough)

## Data Availability

Not applicable.
